# Supplementary material for: The nuclear localization signal of CPSF6 governs post-nuclear import steps of HIV-1 infection
Source: bioRxiv. 2024 Jun 24:2024.06.20.599834. Preprint. [Version 1] doi: 10.1101/2024.06.20.599834 (PMC11230232; doi:10.1101/2024.06.20.599834)
Supplement: Supplement 1 [file NIHPP2024.06.20.599834v1-supplement-1.pdf]

bioRxiv preprint doi: https://doi.org/10.1101/2024.06.20.599834; this version posted June 24, 2024. The copyright holder for this preprint (which was not certified by peer review) is the author/funder, who has granted bioRxiv a license to display the preprint in perpetuity. It is made available under aCC-BY 4.0 International license.

Supporting Information:

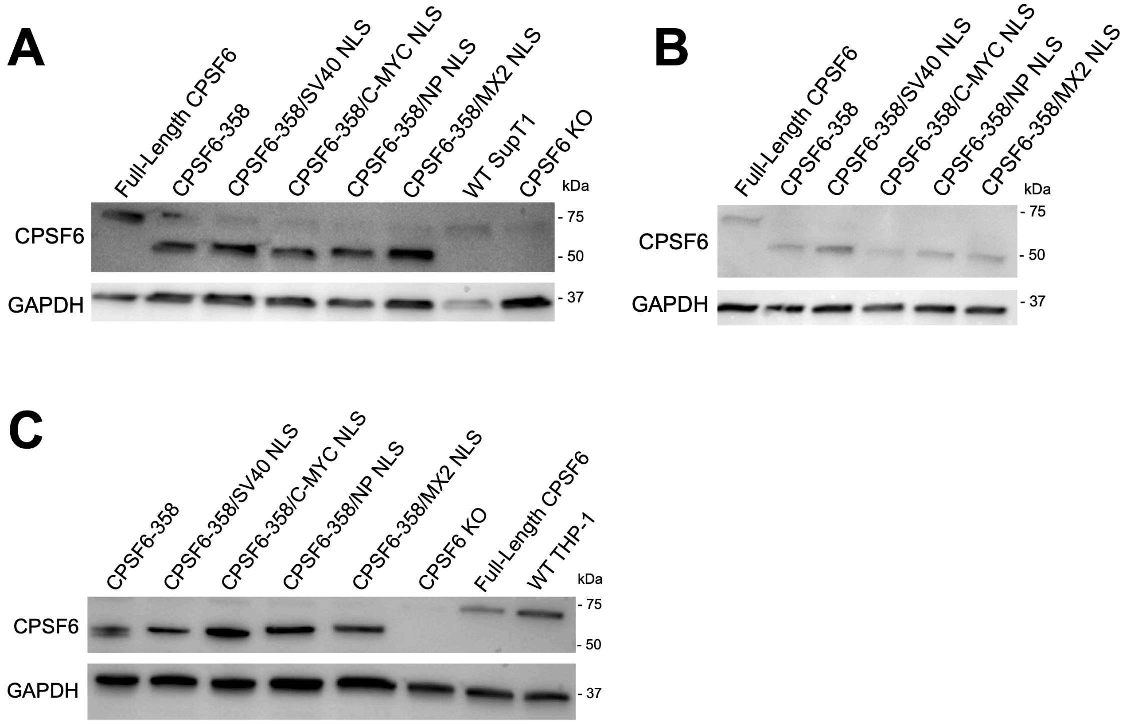

S1 Fig. Chimeric construct expression in T cells and macrophages. Western blot analysis of CPSF6-depleted stably-transduced SupT1 (A), Jurkat (B), and THP-1 (C) cell lines using anti-CPSF6 antibody to detect CPSF6-NLS construct expression following 48 h doxycycline induction. Anti-GAPDH antibody used as loading control.

bioRxiv preprint doi: https://doi.org/10.1101/2024.06.20.599834; this version posted June 24, 2024. The copyright holder for this preprint (which was not certified by peer review) is the author/funder, who has granted bioRxiv a license to display the preprint in perpetuity. It is made available under aCC-BY 4.0 International license.

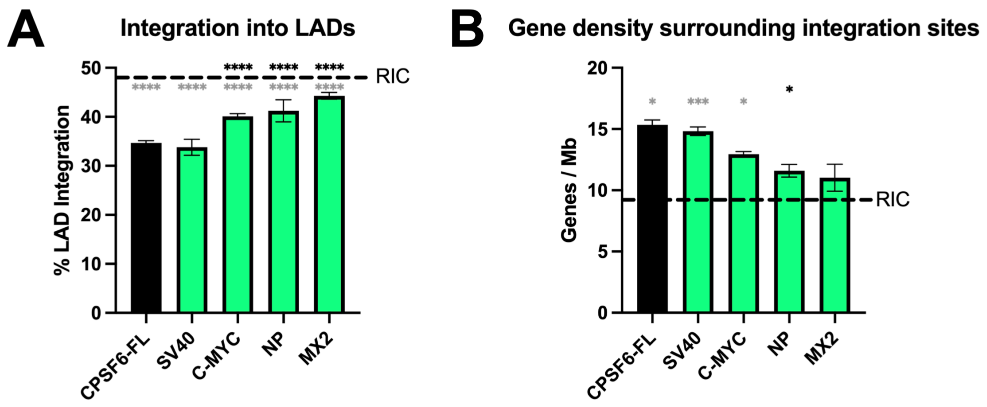

S2 Fig. Integration site targeting metrics. (A) HIV-1 integration targeting with respect to LADs for the indicated cell lines. Fractional LAD targeting in CPSF6-C-MYC, NLS CPSF6-NP NLS, and CPSF6-MX2 NLS-expressing cells was increased significantly compared to cells expressing CPSF6-FL and CPSF6-SV40 NLS. (B) Integration into gene rich regions of chromatin. Integration in gene dense regions in CPSF6-NP NLS cells was reduced significantly compared to CPSF6-FL expressing cells. Integration in NLS CPSF6-NP NLS and CPSF6-MX2 NLS cells was interestingly statistically indistinguishable from the RIC value. * P<0.05, *** P<0.001, **** P<0.0001 (black asterisks, vs CPSF6-FL cells; grey asterisks, vs corresponding RIC).

S1 Table. Integration distributions in CPSF6-NLS chimera HeLa cells[a]

Sample[b]	Unique Sites	% Integration in Genes	% LAD Integration	% SPAD Integration	Gene Density Surrounding Integration Site
C6FL Ctrl	59				
C6FL inf A	3007	69.74	34.25	512	14.94
C6FL inf B	3951	71.40	35.16	745	15.74
SV40 ctrl	28				
SV40 inf A	2236	70.71	32.16	385	15.18
SV40 inf B	1667	69.71	35.45	259	14.48
C-MYC Ctrl	42				
C-MYC inf A	5864	69.24	39.56	752	13.16
C-MYC inf B	4666	67.19	40.63	581	12.72
NP Ctrl	18				
NP inf A	315	59.05	43.49	30	11.08
NP inf B	844	64.45	38.98	98	12.10
MX2 Ctrl	21				
MX2 inf A	429	62.70	43.59	51	12.12

bioRxiv preprint doi: https://doi.org/10.1101/2024.06.20.599834; this version posted June 24, 2024. The copyright holder for this preprint (which was not certified by peer review) is the author/funder, who has granted bioRxiv a license to display the preprint in perpetuity. It is made available under aCC-BY 4.0 International license.

MX2 inf B	496	64.72	44.96	36	9.92
C6KO Ctrl	87				
C6KO inf	2273	62.74	44.13	220	11.50
RIC	112183	45.09	51.20	3178	7.88

[a]Results are from two independent infection experiments (inf A and inf B).
[b]C6FL – full-length CPSF6; C6KO – CPSF6-knockout; RIC – random integration control.
